# Interactive effect of gibberellic acid and NPK fertilizer combinations on ramie yield and bast fibre quality

**DOI:** 10.1038/s41598-017-09584-5

**Published:** 2017-09-06

**Authors:** Sana Ullah, Sumera Anwar, Muzammal Rehman, Shahbaz Khan, Sara Zafar, Lijun Liu, Dingxiang Peng

**Affiliations:** 1 MOA Key Laboratory of Crop Ecophysiology and Farming System in the Middle Reaches of the Yangtze River, College of Plant Science and Technology, Huazhong Agricultural University, Wuhan, 430070 China; 2 Department of Botany, Government College University, Faisalabad, Pakistan

## Abstract

Understanding the effects of different combinations of nitrogen (N), phosphorus (P) and potassium (K) fertilizers and the effects of GA_3_ (gibberellic acid) foliar spray on the fiber quality and yield of ramie are important for maximizing the economic value of these plants. Three pot experiments were conducted using low NPK (140:70:140 kg/ha), normal NPK (280:140:280 kg/ha), and low NPK + GA_3_ (10 mg/L) treatments. In each experiment, following fertilizers were applied: no fertilizer (control), N, P, K, NP, NK, PK, and NPK. Ramie was harvested three times from each plant; ramie grown without fertilizers had significantly lower biomass and yield than plants grown with fertilizers. At both normal and low fertilization rates, application of NPK resulted in greater growth and yield than application of N, P, K, NP, NK, or PK. Unfertilized plants produced the thinnest fibres (22-24 µm), with lowest elongation rate (3.0–3.1%) and breaking strength (22.7–23.3 cN). Fibre yield and fibre quality were improved by application of GA_3_ + fertilizers_._ Maximum fibre yield was obtained at low NPK + GA_3_ treatment, resulting in 65–81% more yield than low NPK alone. GA_3_ with low NPK treatment significantly improved fibre diameter, fibre elongation, and breaking strength compared to both NPK alone and control treatment.

## Introduction

An important aspect of agriculture is the cultivation of plants for food, fiber, biofuel, medicine and other products used to sustain and enhance human life. Agriculture was the key development in the rise of sedentary human civilization, whereby farming of domesticated species created food surpluses that nurtured the development of civilization^1–4^. In response to the current ecological and environmental problems, the textile industry has increased its demand for eco-friendly natural fibres. Additionally, the use of fully biodegradable “green” composites made from vegetable fibres and non-woody plant fibres for paper production may help to mitigate global warming^5^. Bast (phloem) fibres are a considerable source of commercial fibres and are obtained from crops such as *Linum usitatissimum* (flax), *Cannabis saliva* (hemp), *Corchorus capsularis* (jute), *Hibiscus cannabinus* (kenaf), and *Boehmeria nivea* (ramie). Ramie or China grass (*Boehmeria nivea* (L.) Gaud.) is a perennial herbaceous plant, mainly grown in China and other Asian countries. The fibres obtained from ramie plants are the longest known plant fibres in nature and attain a length of more than 550 mm^6, 7^. Ramie fibre has high strength, good durability, moisture absorbance capacity, and high lustre. These characteristics have made ramie fibre suitable for use in the manufacture of a wide variety of textiles and cordage products. Ramie can be blended with other natural and synthetic fibres, including cotton, silk, wool, polyester, and flax^8, 9^. However, despite the remarkable qualities of this fibre, ramie has received comparatively little attention as an important world crop. However, commercial cultivation of this crop has recently increased in countries such as China, Brazil, and the Philippines^10^.

Yield and fibre quality are the most important factors to consider in ramie production. As the bast fibre from ramie is extracted from the outer part of the stem, the fibre yield is dependent on the biomass, length, diameter, and thickness of the stem. Fibre from ramie is normally harvested between three and six times each year with an average annual yield of nearly 1200–1800 kg ha^−1^ of fibre^11^. Due to the plant’s robust growth and biomass production, the fibre yield of ramie is highly dependent on the availability of soil nutrients. According to Hiroce *et al*., ramie plants cannot continue to grow without fertilizers after they reach 60 days of age^12^. The application of fertilizer is crucial for sustaining fibre yield, and optimizing yield requires investigation into suitable fertilization rates^13^.

Growth regulators play an essential role in the biosynthesis of crop fibres, affecting both the elongation rate and quality. The gibberellins (GA) are natural plant growth promoting hormones that cause the elongation of plant cells. Exogenous application of GAs alters plant growth and affects developmental features. Gibberellins exist in various forms and the bioactive forms are GA_1_, GA_3_, GA_4_, and GA_7_. One of these forms, gibberellic acid (GA_3_), promotes growth, especially fibre production and elongation, in hemp, jute, kenaf, cotton, and ramie. The greatest concentrations of GA_3_ are found in those tissues that are elongating the most rapidly, such as stems, petioles, and, in some crops, flower inflorescences^14, 15^. The objective of this project was to evaluate several different combinations of low and normal rates of N, P, and K fertilization and the combined effect of a plant growth regulator and a low rate of fertilization on the subsequent growth, yield and fibre quality of ramie.

## Results

### Influence of treatments on growth

There were three treatment groups: low NPK (N:P:K at 140:70:140 kg ha^−1^), normal NPK (N:P:K at 280:140:280 kg ha^−1^), and low NPK + GA_3_ (gibberellic acid; 10 mg L^−1^). Each treatment group included control, K, P, PK, N, NK, NP, and NPK treatments. The results from a four-way randomized block analysis of variance within treatment groups showed significant effects of harvest time (H) and fertilization on the main growth parameters of ramie (Table 1). However, the interaction between variables (H × N × P × K) was non-significant. All combinations of N, P, and K resulted in greater ramie growth than control treatments, including biomass, number of stems, stem diameter, and stem weight (Tables 2 and 3). Fertilization with NPK resulted in the maximum plant height, biomass, stem weight, and number of stems. The height of plants fertilized with NPK varied significantly between treatment groups:plant height was 29–72% greater than controls for the low NPK group, 31–83% greater than controls for the normal NPK group, and 54–106% greater than controls for the low NPK + GA_3_group. Overall, the maximum height, biomass, and stem diameter of ramie plants were attained by low NPK + GA_3_ plants at the third harvest (H_3_) and the minimums were observed for control plants at the first harvest (H_1_). The overall greatest stem fresh weight was found for low NPK + GA_3_ plants at the second harvest (H_2_) and the maximum number of stems was recorded for low NPK + GA_3_ plants at H_1._

**Table 1:** Four-way randomized block ANOVA evaluating the effect of harvest (H) number, nitrogen, phosphorous, potassium and their interactions on the growth and yield components of ramie.

Source of variation	Plant height cm	Biomass kg	Stem weight	Stem NO.	Stem diameter mm	Raw fiber yield kg	Degummed fiber yield kg
**Low NPK**							
H	***	***	***	***	***	***	***
N	***	Ns	***	***	***	***	***
P	**	***	***	*	*	*	***
K	**	**	***	Ns	*	**	***
N × P × K	**	Ns	Ns	*	***	Ns	**
H × N × P × K	Ns	*	Ns	Ns	Ns	Ns	**
**Normal NPK**							
H	***	***	***	***	***	***	***
N	***	***	***	***	**	***	***
P	**	***	***	*	***	***	***
K	**	***	***	***	***	***	***
N × P × K	***	Ns	Ns	Ns	**	Ns	Ns
H × N × P × K	Ns	Ns	Ns	Ns	Ns	*	Ns
**Low NPK** + **GA**_**3**_							
H	***	***	***	**	***	***	***
N	***	***	***	***	***	***	***
P	***	***	***	***	***	***	***
K	***	***	***	***	***	***	***
N × P × K	**	Ns	Ns	Ns	**	Ns	Ns
H × N × P × K	Ns	Ns	Ns	Ns	Ns	*	Ns

Ns, non-significant; *, significant at *p* < 0.05; **, significant at *p* < 0.01; and ***, significant at *p* < 0.001.

**Table 2:** Plant height and biomass of ramie under different treatments at three harvests (H_1_, H_2_, and H_3_).

Treatments	Plant height (cm)			Plant biomass (kg)		
	H_1_	H_2_	H_3_	H_1_	H_2_	H_3_
**Low NPK (kg/ha)**						
N_0_P_0_K_0_ (control)	49.0b	49.0c	60.4b	0.11c	0.27c	0.13c
N_0_P_0_K_140_	58.7ab	73.0b	86.2a	0.18bc	0.54ab	0.36ab
N_0_P_70_K_0_	60.3a	76.7ab	83.4a	0.18bc	0.47abc	0.23bc
N_0_P_70_K_140_	67.7a	88.7a	71.4ab	0.25a	0.35bc	0.44ab
N_140_ P_0_ K_0_	60.3a	89.0a	81.6a	0.14c	0.31c	0.27abc
N_140_ P_0_K_140_	61.3a	79.3ab	79.3a	0.22ab	0.33bc	0.31abc
N_140_P_70_ K_0_	59.3ab	82.7ab	79.3a	0.24ab	0.42abc	0.49a
N_140_P_70_K_140_	63.3a	84.3ab	88.3a	0.26a	0.56a	0.46a
Mean	60.0 A	77.7 A	78.7 A	0.19 C	0.41 A	0.34B
**Normal NPK (kg/ha)**						
N_0_ P_0_ K_0_ (control)	56.3c	53.7c	63.3e	0.08d	0.18c	0.11d
N_0_ P_0_ K_280_	64.3abc	88.0ab	88.0abc	0.26bc	0.45ab	0.34c
N_0_ P_140_ K_0_	66.7abc	88.0ab	93.7ab	0.29abc	0.46ab	0.44bc
N_0_ P_140_K_280_	73.0a	77.0b	71.7de	0.33ab	0.39b	0.58ab
N_280_P_0_ K_0_	62.3bc	99.0a	97.3a	0.25c	0.48ab	0.47abc
N_280_P_0_ K_280_	70.3ab	84.3b	83.7bc	0.33abc	0.53ab	0.62a
N_0_ P_140_ K_0_	69.0ab	86.3ab	0.7cd	0.35a	0.61a	0.49abc
N_0_ P_140_K_280_	74.0a	98.3a	96.0a	0.33ab	0.58a	0.59ab
Mean	67.0B	84.3A	84.3A	0.28B	0.46A	0.46A
**Low NPK** + **GA**_**3**_						
N_0_ P_0_ K_0_ (control)	54.7b	66.0c	58.0e	0.11e	0.24e	0.14c
N_0_ P_0_ K_140_ + GA_3_	79.3a	92.7b	96.0bcd	0.30d	0.52bcd	0.48b
N_0_ P_70_ K_0_ + GA_3_	79.0a	94.3ab	88.3d	0.31 cd	0.64abc	0.52ab
N_0_P_70_K_140_ + GA_3_	82.3a	105.0a	102.0bc	0.35bc	0.65abc	0.56ab
N_140_P_0_ K_0_ + GA_3_	78.7a	90.7b	91.7 cd	0.32bcd	0.38de	0.57ab
N_140_P_0_ K_140_ + GA_3_	87.3a	92.7b	106.7b	0.45a	0.68a	0.66ab
N_140_P_70_K_0_ + GA_3_	81.3a	91.7b	94.7bcd	0.36b	0.49 cd	0.71a
N_140_P_70_K_140_ + GA_3_	86.0a	101.3ab	119.7a	0.44a	0.67ab	0.73a
Mean	78.6B	91.8A	94.6A	0.33B	0.53A	0.55A

The low NPK (N:P:K at 140:70:140 kg ha^−1^), normal NPK (N:P:K at 280:140:280 kg ha^−1^) and low NPK + GA_3_ (gibberellic acid; 10 mg L^−1^) treatment groups. Each of the three treatment groups were further subdivided into (Control, K, P, PK, N, NK, NP, and NPK) treatments.

Plants were harvested on June 20 (1st harvest H1), August 10 (2nd harvest H2), and October 10 (3rd harvest H3), 2015, of ramie, respectively. Data followed by different lowercase letters (a, b, c) in the same column indicate statistically significant differences within a harvest; values followed by different uppercase letters (A, B, C) in the same row indicate significant difference between harvests at p < 0.05 based on LSD test.

**Table 3:** Stem weight, number of stem and stem diameter of ramie plants under the low NPK, normal NPK and low NPK + GA_3_ treatment groups at three harvests (H_1_, H_2_, and H_3_).

Treatments	Stem weight (g)			NO of stem (plant^−1^)			Stem diameter (mm)		
	H_1_	H_2_	H_3_	H_1_	H_2_	H_3_	H_1_	H_2_	H_3_
**Low NPK**									
N_0_P_0_K_0_ (control)	22.7b	104.3b	47.7d	2.67b	2.33c	2.67b	4.74c	5.93b	5.93c
N_0_P_0_K_140_	70.7a	180.3ab	175.3abc	7.00a	5.67ab	3.67ab	6.29b	7.77a	8.97a
N_0_P_70_K_0_	79.7a	190.3ab	110.3bcd	6.00a	5.00b	4.00ab	6.73ab	8.20a	8.39ab
N_0_P_70_K_140_	104.0a	166.3ab	185.7ab	6.33a	5.67ab	4.00ab	6.53ab	7.17ab	7.32bc
N_140_P_0_K_0_	62.0ab	175.3ab	90.7 cd	6.67a	7.00ab	5.33a	6.39ab	7.93a	8.42ab
N_140_P_0_K_140_	78.3a	182.7ab	160.7abc	6.00a	6.33ab	5.00a	7.42a	8.20a	8.06ab
N_140_P_70_K_0_	86.3a	200.7ab	211.0a	7.00a	7.33a	5.67a	6.49ab	8.27a	8.80ab
N_140_P_70_K_140_	87.7a	264.0a	249.0a	7.00a	5.67ab	5.33a	7.20ab	8.03a	8.89a
Mean	73.9B	183.0A	153.8A	6.1A	5.6A	4.5B	6.5B	7.7A	8.1A
**Normal NPK**									
N_0_P_0_K_0_ (control)	29.7c	68.3b	42.3e	3.33c	3.33c	2.33c	4.76b	5.70b	6.21b
N_0_P_0_K_280_	110.0b	197.3a	139.7d	6.00ab	5.67abc	4.67abc	7.00a	8.47a	8.66a
N_0_P_140_K_0_	111.7b	198.0a	153.0d	6.67a	4.33bc	3.33bc	7.84a	8.27a	9.22a
N_0_P_140_K_280_	169.3a	171.3ab	266.0ab	6.00ab	4.67abc	5.00ab	7.70a	8.60a	9.45a
N_280_P_0_K_0_	121.7ab	205.0a	187.3cd	4.67bc	6.33ab	4.67abc	6.94a	8.27a	8.81a
N_280_P_0_K_280_	157.7ab	233.7a	235.7abc	6.67a	7.33a	6.00a	7.60a	8.57a	9.09a
N_280_P_140_K_0_	159.7ab	257.7a	204.3bcd	7.00a	7.33a	4.67abc	7.40a	8.20a	9.24a
N_280_P_140_K_280_	168.0a	200.0A	190.8A	7.67a	7.33a	5.67ab	8.18a	8.60a	9.30a
Mean	128.5B	268.7a	298.3a	6.0A	5.8A	4.5B	7.18 C	8.08B	8.75 A
**Low NPK** + **GA**_**3**_									
N_0_P_0_K_0_ (control)	27.0c	125.3c	54.3d	3.67d	3.33e	2.67e	4.44d	5.77d	6.18c
N_0_P_0_K_140_+GA_3_	127.6ab	250.0b	213.0c	6.67bc	5.67d	4.67d	6.50c	8.33c	8.25b
N_0_P_70_K_140_+GA_3_	121.7b	268.3ab	223.3bc	6.00c	7.00bcd	5.33cd	7.39abc	9.27ab	9.62ab
N_0_P_70_K_0_+GA_3_	172.7ab	330.3a	234.0bc	8.33a	7.67ab	6.00bcd	8.47a	8.97abc	10.05a
N_140_P_0_K_0_+GA_3_	146.0ab	233.7bc	287.3abc	6.67bc	6.00cd	7.00ab	7.39abc	8.50bc	9.61ab
N_140_P_0_K_140_+GA_3_	184.3ab	342.7ab	294.3ab	8.00ab	7.33ab	8.33a	8.16ab	9.47a	10.28a
N_140_P_70_K_0_+GA_3_	178.0ab	280.0ab	357.3a	7.67ab	7.33abc	6.33bc	7.01bc	9.20abc	9.64ab
N_140_P_70_K_140_+GA_3_	188.0a	368.7a	330.7ab	8.67a	8.67a	7.33abc	8.37a	9.13abc	9.90ab
Mean	143.2B	274.9A	249.3A	6.96A	6.62A	5.96B	7.22C	8.58B	9.19A

The low NPK (N:P:K at 140:70:140 kg ha^−1^), normal NPK (N:P:K at 280:140:280 kg ha^−1^) and low NPK + GA_3_ (gibberellic acid; 10 mg L^−1^) treatment groups. Each of the three treatment groups were further subdivided into (Control, K, P, PK, N, NK, NP, and NPK) treatments.

Plants were harvested on June 20 (1st harvest H_1_), August 10 (2nd harvest H_2_), and October 10 (3rd harvest H_3_), 2015, of ramie, respectively. Data followed by different lowercase letters (a, b, c) in the same column indicate statistically significant differences within a harvest; values followed by different uppercase letters (A, B, C) in the same row indicate significant difference between harvests at p < 0.05 based on LSD test.

### Influence of treatments on fibre yield

**Figure 1:**
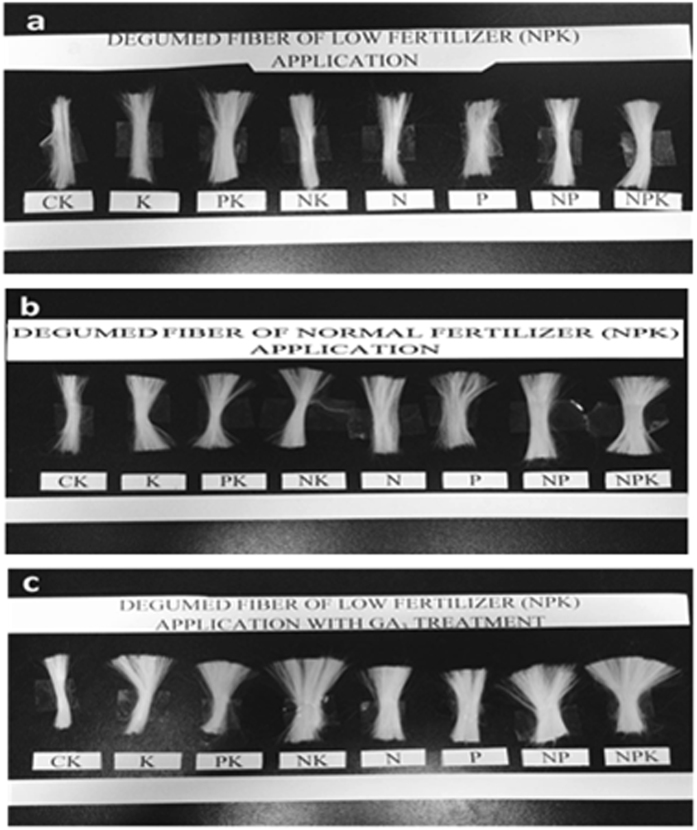
Degummed ramie bast fibres obtained from plants treated with (**a**) a low rate of NPK fertilization (N:P:K at 140:70:140 kg ha^−1^), (**b**) a normal rate of NPK fertilization (N:P:K at 280:140:280 kg ha^−1^), and (**c**) a low rate of NPK fertilization + gibberellic acid (10 mg L^−1^). CK represents controls. K, P, and N indicate fertilization with potassium, phosphorous, and nitrogen, respectively.

Fibre yield was significantly greater for fertilizer treatments than for controls (Table 4, Fig. 1). However, the interactions between harvest and fertilizer types (H × N × P × K) remained non-significant, with the exceptions of biomass in the low NPK treatment group and raw fibre yield in the normal NPK and low NPK + GA_3_ treatment groups.

**Table 4:** Raw fiber yield and degummed fiber yield from ramie under different the low NPK, normal NPK and low NPK + GA_3_ treatment groups at three harvests (H_1_, H_2_, and H_3_).

Treatments	Raw fiber yield (g)			Degummed fiber yield (g)		
	H_1_	H_2_	H_3_	H_1_	H_2_	H_3_
**Low NPK**						
N_0_ P_0_ K_0_ (control)	10.0b	35.3c	17.0d	2.14d	5.0c	2.37c
N_0_ P_0_ K_140_	29.7ab	91.7a	42.3bd	4.28c	13.3ab	4.78b
N_0_ P_70_ K_0_	31.7ab	71.0ab	33.0 cd	4.94bc	10.7ab	5.73b
N_0_ P_70_ K_140_	37.7a	60.7bc	64.7ac	5.50b	9.3bc	8.30a
N_140_ P_0_ K_0_	33.7a	80.0ab	62.0ac	5.25b	10.0bc	4.83b
N_140_ P_0_K_140_	35.7a	68.7ab	73.7ab	4.85bc	8.7bc	8.84a
N_140_ P_70_ K_0_	36.0a	76.7ab	83.7a	4.92bc	10.3b	8.87a
N_140_ P_70_ K_140_	47.0a	81.0ab	79.0ab	6.70a	15.7a	9.80a
Mean	32.7 C	70.6 A	56.9B	4.8 C	10.4 A	6.7B
**Normal NPK**						
N_0_ P_0_ K_0_ (control)	15.7c	30.3c	17.7d	2.38c	5.0c	2.61d
N_0_ P_0_ K_280_	41.7bc	81.3ab	49.7c	8.65ab	11.3b	6.16c
N_0_ P_140_ K_0_	46.3ab	87.0ab	55.0c	6.86b	12.3b	6.67bc
N_0_ P_140_K_280_	64.3ab	69.3abc	95.0a	8.81ab	12.0b	10.72a
N_280_ P_0_ K_0_	48.0ab	76.7ab	60.7bc	6.95b	11.7b	6.32c
N_280_ P_0_ K_280_	55.3ab	61.7bc	88.7ab	8.32ab	13.0ab	9.10ab
N_280_ P_140_ K_0_	70.3a	99.3ab	79.7ac	9.54a	15.0ab	9.15ab
N_280_ P_140_ K_280_	66.7ab	109.7a	108.7a	9.42a	17.3a	10.41a
Mean	51.0B	76.9 A	69.4A	7.62B	12.2A	7.6B
**Low NPK** + **GA**_**3**_						
N_0_ P_0_ K_0_ (control)	13.7e	49.0d	27.0d	2.27 f	6.33d	3.74b
N_0_ P_0_ K_140_ + GA_3_	51.7d	95.0c	82.3c	8.27d	13.3c	9.56a
N_0_ P_70_ K_0_ + GA_3_	48.0d	98.3bc	84.7c	6.95e	14.3c	9.35ab
N_0_ P_70_ K_140_ + GA_3_	78.0b	125.7ab	106.7c	10.44bc	17.7bc	11.21a
N_140_ P_0_ K_0_ + GA_3_	66.3c	92.3c	85.0c	9.22 cd	13.7c	10.78a
N_140_ P_0_ K_140_ + GA_3_	90.7a	148.0a	108.7c	11.42ab	21.0ab	11.69a
N_140_ P_70_ K_0_ + GA_3_	82.7ab	103.3bc	149.3a	11.11ab	15.0c	12.92a
N_140_ P_70_ K_140_ + GA_3_	88.0ab	147.3a	130.3b	11.98a	22.3a	12.99a
Mean	64.9C	107.4A	96.7B	8.96C	15.5A	10.3B

The low NPK (N:P:K at 140:70:140 kg ha^−1^), normal NPK (N:P:K at 280:140:280 kg ha^−1^) and low NPK + GA3 (gibberellic acid; 10 mg L^−1^) treatment groups. Each of the three treatment groups were further subdivided into (Control, K, P, PK, N, NK, NP, and NPK) treatments.

Plants were harvested on June 20 (1st harvest H1), August 10 (2nd harvest H2), and October 10 (3rd harvest H3), 2015, of ramie, respectively. Data followed by different lowercase letters (a, b, c) in the same column indicate statistically significant differences within a harvest; values followed by different uppercase letters (A, B, C) in the same row indicate significant difference between harvests at p < 0.05 based on LSD test.

The fresh and degummed fibre yields were greatest for the low NPK + GA_3_ treatment group, followed by the normal NPK and low NPK treatment groups. For all treatment groups, the fresh fibre yield and degummed fibre yield were highest at H_2_, followed by those at H_3_ and H_1_. The fibres with the lowest fresh and dry weights were from control plants. In the low and normal NPK treatment groups, the combined application of NPK resulted in higher fresh and degummed fibre yield than application of K, P, PK, N, NK, or NP. However, in the low NPK + GA_3_ treatment group, the highest fresh yield was recorded for NK + GA_3_ treatment_._

### Influence of treatments on fibre quality traits

**Figure 2:**
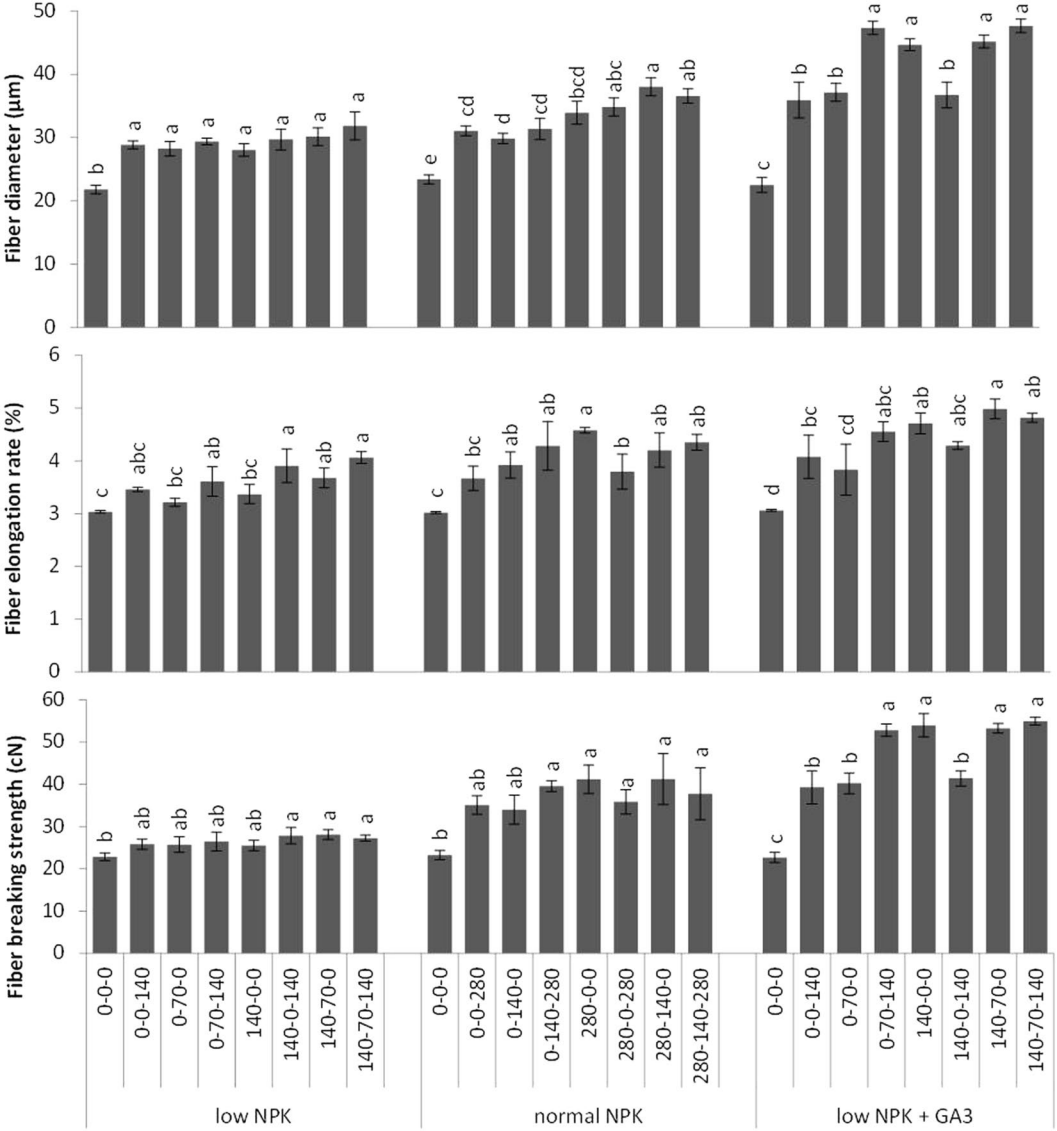
Diameter, elongation rate, and breaking strength of ramie fibre under different NPK combinations for low NPK (N:P:K at 140:70:140 kg ha^−1^), normal NPK (N:P:K at 280:140:280 kg ha^−1^) and low NPK + GA_3_ (gibberellic acid; 10 mg L^−1^) treatment groups (*n* = 3 ± SE). Different letters (**a**,**b**,**c**) indicate statistically significant differences among treatments at p < 0.05 based on LSD tests.

The measures of fibre yield and quality, including the fibre breaking strength, elongation rate, and diameter, were positively affected by fertilizer treatment (Fig. 2). Fibre diameter increased with the application of fertilizers. The thinnest fibres were from unfertilized plants (22–24 µm), and the thickest fibres were from plants in the low NPK + GA_3_ treatment group that received the NP treatment (47.6 µm)_._ The lowest elongation rate was observed for fibres from unfertilized plants. The maximum elongation rate was observed for fibres from plants in the low and normal NPK treatment groups that received NK treatment and for fibres from plants in the low NPK + GA_3_ treatment group that received NP treatment.

The lowest breaking strength was observed for fibres from unfertilized plants, and the highest breaking strength was observed for fibres from plants in the low NPK + GA_3_ treatment group that received NK treatment, followed by those from the low NPK treatment group that received N and NP treatments.

## Discussion

In the present study, ramie responded positively to NPK fertilizers and foliar application of GA. Ramie fibres mainly consist of secondary phloem fibres and the economic value of this plant is based on the amount of fibre produced. Increasing the plant height, biomass, stem diameter, stem weight, and number of stems per plant ultimately increases the bast fibre yield of ramie. Among various combinations of N, P, and K fertilizers tested, the combined application of NPK was the most effective in increasing the fibre yield and fibre quality traits of ramie in all pot experiments. It is well known that N, P, and K are essential nutrients for plant growth. These nutrients are utilized in large amounts because N is an essential component of nucleic acid and protein synthesis, P is used in energy compounds (ATP and ADP) and nucleic acids, and K helps in the transport of water and nutrients through the xylem and is involved in the activation of many enzymes^16^. In the present study, fertilizer treatments that did not include N, such as the K, P, and PK treatments, produced shorter plants with less biomass and stem weight than the NK, NP, and NPK treatments. Ullah *et al*., has also reported that treatment with combined NPK (150–75–150 kg ha^−1^) maximizes plant characteristics that affect ramie fiber yield^17^. Among the essential plant nutrients, N plays the most important role in improving agricultural production^17, 18^. N application promotes the growth and fiber yield of ramie by increasing plant chlorophyll, soluble protein, and proline content; reducing MDA content; and enhancing gas exchange parameters and antioxidant enzyme activity^19^. It is possible, however, that it is the interaction between nutrients, rather than their absolute concentration, that is most important for maximizing fertilizer use efficiency^20^.

In the present study, ramie plants that received a normal rate of NPK fertilization attained greater height, biomass, number of stems, and stem weight than those that received a low rate of NPK fertilization. The recommended fertilization rates for ramie vary with the soil type, growing conditions, and ramie genotype. For example, 90:60:60 kg ha^−1^ N:P:K is recommended for ramie growth in clay loam soil^21^.

As the stems of ramie plants are the main source of fibres, an increase in stem biomass and diameter results in increased fibre yield^17^. In the present study, the treatments that resulted in the lowest number, weight, and diameter of stems (controls and fertilizer treatments that did not contain N) also resulted in the lowest raw and degummed fibre yield. Similarly, treatments that resulted in the maximum number, weight, and diameter of stems (NPK, NP, and NK treatments) also resulted in the highest fibre yields. These results are in-line with previous reports of a linear relationship between yield measures, such as dry yield, total aboveground biomass and bast fibres, and plant characteristics, such as stem number, plant height and stem basal diameter^22^.

In the present study, harvest time also significantly affected the production of ramie fibre. The second harvest (H_2_) was the most productive, resulting in the greatest fibre yield and stem biomass. This contrasts with results reported by Angelini and Tavarini, who found that higher and thicker stems, with higher bast fibre production per hectare, were obtained from the first ramie harvest than from subsequent harvests^22^.

In the present study, the application of N in combination with P, K, or PK resulted in the highest quality fibres. Fibre breaking strength was increased significantly with fertilizer application and the maximum breaking strength was recorded for fibres from plants in the low and normal NPK treatment groups that received NP treatment. Breaking strength did not increase further by the addition of K. The maximum fibre diameter was obtained for plants in the low NPK group that were treated with NPK and plants in the normal NPK group that were treated with NP. These results contrast with those of Liu *et al*., who concluded that application of N to ramie plants had the greatest effect on growth and fibre yield, whereas supplemental K had discernible effects on fibre quality^15^.

The addition of GA_3_ to fertilized plants in the present study enhanced all recorded growth traits, such as plant height, biomass, stem weight, stem diameter, and the number of stems. High IAA/low GA_3_ concentrations have been shown to have an inhibitory effect on stem elongation, whereas low IAA/high GA_3_ concentrations promote rapid internode elongation^23^. GA_3_ promotes stem elongation by increasing the physiological levels of auxin, either by increasing auxin production or decreasing the destruction of auxin^24^. Spraying ramie with GA may also promote growth and yield by increasing endogenous GA content, eliminating oxidative stress, and maintaining cellular integrity^25^.

We found that the application of GA_3_ to plants resulted in greater production of fibre than fertilizer alone, regardless of the rate of fertilization. The observed increase in fibre yield with the application of GA_3_ can be attributed to improved growth, development of chloroplasts, and intensification of photosynthetic efficiency^26^. Plants treated with GA_3_ had greater stem weight, more bark, and less wood deposition than plants not treated with GA_3_. These are all desirable features for bast-producing plants.

GA affects the differentiation of primary phloem fibre and increases the length of bast fibres by increasing internode length. In *C. blumei*, high levels of GA_3_ result in long phloem fibres with thin walls and the length of differentiating internodes is correlated with the length of primary phloem fibres^23^. The increase in the length of fibres treated with GA_3_ in the present study is likely associated with the observed increase in plant height and with increases in intermodal length.

In addition to relatively long fibres, plants in the low NPK + GA_3_ treatment group that were treated with NPK had fibres that were greater in diameter than plants in the low and normal NPK treatment groups that were treated with NPK. Fibre elongation rate was also maximized by spraying with GA_3_ and fertilizing with NPK. Similarly, in transgenic kenaf and populus trees that over express gibberellic acid, the increased GA has a positive impact on fibre number, length, diameter, and wall thickness^27^.

The breaking strength of fibres from plants in the low NPK + GA_3_ treatment group that were treated with NPK was greater than that for fibres from control plants and those treated with NPK alone. The strength of fibres was likely increased by increases in their length and diameter. The flexural strength of hemp fibres decreases significantly with decreasing fibre length^28^. Similarly, long okra fibres are stronger than short fibres because unlike long cells, short fibre cells require many weak connecting points in order to form fibre strands^29^. According to Withanage *et al*., enhanced bioactive GA is extremely important for increasing the length of kenaf fibre and can be obtained by over expressing the *Arabidopsis thaliana* gibberellic acid 20 oxidase gene (AtGA20ox) in transgenic kenaf plants^27^.

The quantity and quality of ramie bast fibre were significantly affected by harvest, rate of NPK fertilizer, and foliar application of GA_3_. Plant height, biomass, stem weight, stem diameter, number of stems, fibre yield, fibre elongation rate, fibre diameter, and fibre breaking strength were improved by fertilizer application. The application of NPK at a normal rate of fertilization was more successful in enhancing these traits than application of NPK at a low rate of fertilization or the application of N, P, or K alone. The maximum fibre yield and fibre quality traits were observed for plants treated with a low rate of NPK fertilization and foliar application of GA_3_. Therefore, spraying ramie plant canopies with GA_3_ and providing NPK fertilizer at a low rate can enhance fibre yield while reducing the requirement for normal fertilizer doses.

## Materials and Methods

A pot experiment was carried out in a greenhouse at Huazhong Agricultural University, Wuhan, China. Rhizome segments (15 cm) obtained from the roots of the normal yield biannual ramie cultivar, Huazhu-5, were obtained from the experimental base at Huazhong Agricultural University. Pots (60 cm diameter) were filled with soil containing 11 g kg^−1^ of organic matter, 40 g kg^−1^ total N, 0.18% total P, and 60 g kg^−1^ total K with EC: 2 dS cm^−1^ and pH: 5.8. The rhizome segments were planted in the pots on March 25, 2015. Plants were harvested on June 20 (H_1_), August 10 (H_2_), and October 1 (H_3_), 2015, by cutting stems 10 cm above the soil.

### NPK fertilizer and exogenous application of GA3

**Figure 3:**
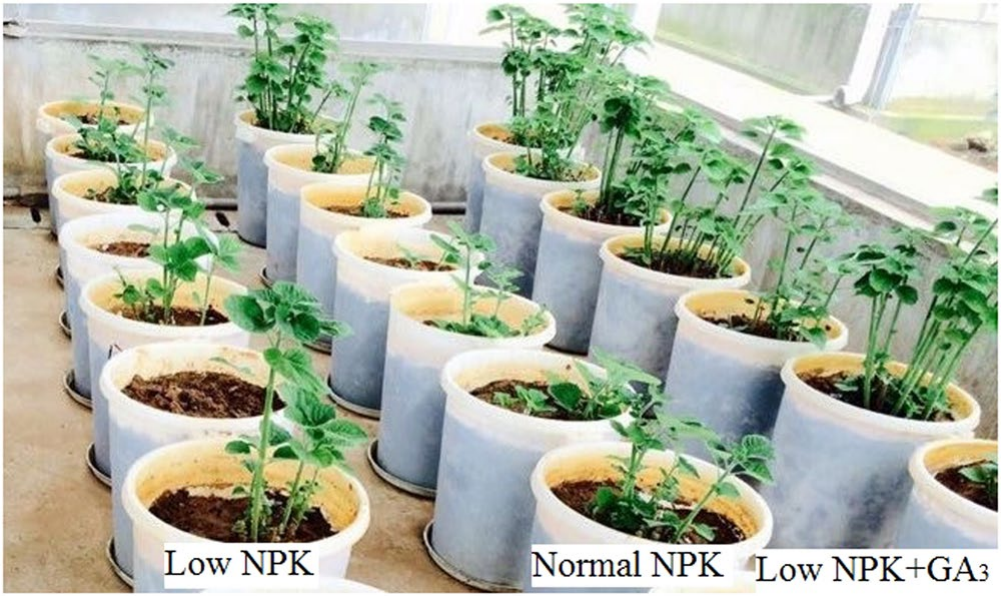
Growing ramie in a greenhouse under different fertilizer conditions: low NPK (N:P:K at 140:70:140 kg ha^−1^), normal NPK (N:P:K at 280:140:280 kg ha^−1^), and low NPK + GA_3_ (gibberellic acid; 10 mg L^−1^).

The prepared pots were separated into low NPK, normal NPK and low NPK + GA_3_ treatment groups (Fig. 3). Each of the three treatment groups was further subdivided into K, P, PK, N, NK, NP, and NPK treatments. In the low NPK groups, fertilizer concentrations were 140, 70, and 140 kg ha^−1^ for N, P, and K respectively. In the normal NPK group, fertilizer concentrations were 280, 140, and 280 kg ha^−1^ for N, P, and K, respectively. Controls received no fertilizer. P was applied as a single dose in the form of calcium super phosphate (14% P_2_O_5_) at planting. N, in the form of urea (46% N), and K, in the form of potassium chloride (54% K_2_O), were applied in three doses: at planting (40%), in June (30%) after the first harvest, and in August (30%) after the second harvest. For the NPK + GA_3_ treatment group (n = 28), 10 mg L^−1^ GA_3_ was sprayed over the canopy three times. The first dose (50%) was sprayed in April (10 days after planting), and subsequent doses were sprayed 10 days after each harvest, with 30% sprayed in June and 20% sprayed in August. Each treatment was replicated four times, arranged in a randomized complete block design.

### Plant growth and fibre evaluation

Before each harvest, the effective number of stems in each pot was counted and plant height was measured from the root neck to the upper most part of the stalk. After each harvest, the remaining plants in each pot were allowed to re-grow until the next harvest. Stem diameter (mm) was measured at a height of 15 cm above soil surface using a digital Vernier calliper (ST22302, SG tools, Hangzhou, China). Plant biomass was measured by weighing both stems and leaves and stems were weighed again separately after removing all leaves. The fibre layer of each stem was decorticated (peeled from the pith), the epidermis was removed, and raw fibres were weighed to calculate fibre yield. Then, 20 g of decorticated fibre was boiled for 1 h in an Erlenmeyer flask containing 100 mL of degumming solution (1 g NaOH and 0.05 g EDTA). The degummed fibres were bleached with 2% H_2_O_2_ and 0.1% Tween-80 for 1 h at 94 °C in a water bath, washed with distilled water, and dried and combed (Fig. 2). Fibre diameter (µm) was measured using a computerized fibre fineness tester (Model No. YG002C, Changzhou, China) connected to an optical microscope. Fibre breaking strength (centi newtons, cN) and elongation rate (%) were determined using a fibre strength tester (YG004, Nantong Hongda Experiment Instruments, Qidong, China), following the Chinese National Standards (GB 5882–86).

### Statistical analysis

All data were subjected to analysis of variance (ANOVA) using the statistical software CoStat Version 6.303 (CoHort, USA). The effects of harvest time (H), nitrogen (N), phosphorus (P), potassium (K), and their interactions (H × N × P × K) were analysed by a four-way randomized block ANOVA. Means and standard errors were calculated and graphs were prepared using Microsoft Office Excel (2007).

## References

[1] Ercisli S (2009). Apricot culture in Turkey. Sci. Res. Essays..

[2] Erturk Y, Ercisli S, Haznedar A, Cakmakci R (2010). Effects of plant growth promoting rhizobacteria (PGPR) on rooting and root growth of kiwifruit (*Actinidia deliciosa*) stem cuttings. Biol. Res..

[3] Saridas MA, Kafkas NE, Zarifikhosroshahi M, Bozhaydar O, Kargi SP (2016). Quality traits of green plums (*Prunus cerasifera* Ehrh.) at different maturity stages. Turk. J. Agric. For..

[4] Yazici K, Sahin A (2016). Characterization of pomegranate (*Punica Granatum* L.) hybrids and their potential use in further breeding. Turk. J. Agric. For..

[5] Goda K, Sreekala MS, Gomes A, Kaji T, Ohgi J (2006). Improvement of plant based natural fibers for toughening green composites-effect of load application mercerization of ramie fibers. Compos. Part A: Appl. Sci. Manuf..

[6] Aldaba VC (1927). The structure and development of the cell wall in plants I. Bast fibers of Boehmeria and Linum. Am. J. Bot..

[7] Lev-Yadun S (2010). Plant fibers: Initiation, growth, model plants, and open questions. Russ. J. Plant Physio..

[8] Kalita BB, Gogoi N, Kalita S (2013). Properties of ramie and its blends. Int. J. Eng. Res. Gen. Sci..

[9] Mitra, S. *et al*. Ramie: The Strongest Bast fibre of Nature. *Technical Bulletin No. 8, Central Research Institute for Jute and Allied Fibres, ICAR, Barrackpore, Kolkata*−120. 38 (2013).

[10] Jose S, Rajna S, Ghosh P (2017). Ramie fibre processing and value addition. Asian J. Tex..

[11] Sen T, Reddy HJ (2011). Various industrial applications of hemp, kinaf, flax and ramie natural fibres. Int. J. Innov. Man. Technol..

[12] Hiroce R, Benatti Júnior R, Fujiwara M, Paulo EM (1985). Nutrient uptake by ramie ‘Miyasaki’ grown under greenhouse conditions. Bragantia..

[13] Subandi M (2012). The effect of fertilizers on the growth and the yield of ramie (*Boehmeria nivea* L. Gaud). Asian J. Agric. Rural Dev..

[14] Ayala-Silva T, Akin DE, Foulk J, Dodd RB (2005). Effect of growth regulators on yield and fiber quality and quantity in flax (*Linum usitatissimum* L.). Plant Growth Regul. Soc. Am..

[15] Liu LJ, Chen HQ, Dai XB, Hui WANG, Peng DX (2012). Effect of planting density and fertilizer application on fiber yield of ramie (*Boehmeria nivea*). J. Integr. Agric..

[16] Pal P, Yadav K, Kumar K, Singh N (2016). Effect of gibberellic acid and potassium foliar sprays on productivity and physiological and biochemical parameters of parthenocarpic cucumber cv. ‘seven star F1’. J. Hortic. Res..

[17] Ullah S (2016). Effects of fertilization on ramie (*Boehmeria nivea* L.) growth, yield and fiber quality. Sustainability..

[18] Khan S (2017). Optimization of nitrogen rate and planting density for improving yield, nitrogen use efficiency, and lodging resistance in Oilseed Rape. Front. Plant Sci..

[19] Huang C (2014). Effects of nitrogen on ramie (*Boehmeria nivea*) hybrid and its parents grown under field conditions. J. Agric. Sci..

[20] Aulakh MS, Malhi SS (2005). Interactions of nitrogen with other nutrients and water: effect on crop yield and quality, nutrient use efficiency, carbon sequestration, and environmental pollution. Adv. Agron..

[21] Cabangbang RP (1978). Fiber yield and agronomic characters of ramie as affected by plant density and fertilizer level. Philipp. J. Crop Sci..

[22] Angelini LG, Tavarini S (2013). Ramie (*Boehmeria nivea* L.) as a potential new fibre crop for the Mediterranean region: Growth, crop yield and fibre quality in a long-term field experiment in Central Italy. Ind. Crops Prod..

[23] Aloni R (1979). Role of auxin and gibberellin in differentiation of primary phloem fibers. Plant Physiol..

[24] Ross JJ, O’Neill DP, Rathbone DA (2003). Auxin-gibberellin interactions in pea: integrating the old with the new. J. Plant Growth Regul..

[25] Liu T (2013). Morphological and physiological changes of ramie (*Boehmeria nivea* L. Gaud) in response to drought stress and GA_3_ treatment. Russ. J. Plant Physiol..

[26] Yuan L, Xu DQ (2001). Stimulation effect of gibberellic acid short-term treatment on the photosynthesis related to the increase in Rubisco content in broad bean and soybean. Photosynth. Res..

[27] Withanage SP (2015). Over expression of Arabidopsis thaliana gibberellic acid 20 oxidase (AtGA20ox) gene enhance the vegetative growth and fiber quality in kenaf (*Hibiscus cannabinus* L.) plants. Breed. Sci..

[28] Shibata S, Fukumoto I, Cao Y (2006). Effects of fiber compression and length distribution on the flexural properties of short kenaf fiber-reinforced biodegradable composites. Polym. Compos..

[29] Fathima MU, Balasubramanian AR (2006). Effect of plant growth regulators on the quality of bast fibres in *Abelmoschus esculentus* (Linn.) Moench. Acta Bot. Croat..

